# Supplementary material for: Splice-Junction-Based Mapping of Alternative Isoforms in the Human Proteome
Source: Cell Rep. Author manuscript; Available in PMC 2020 Jan 15. (PMC6961840; doi:10.1016/j.celrep.2019.11.026)
Supplement: 2 [file NIHMS1546469-supplement-2.zip › DF1/PXD006675/RightAtrium/RightAtrium_11_TTN_RVEAEPAEEVTIMEEK.pdf]

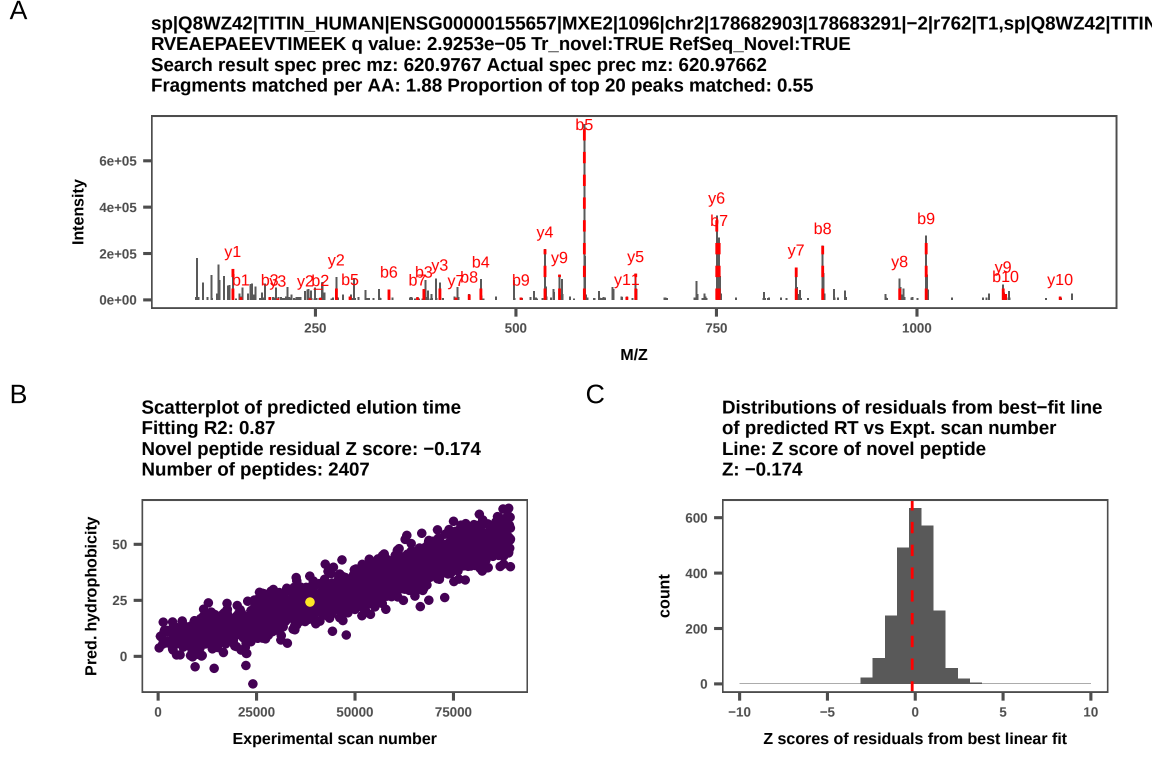
A
sp|Q8WZ42|TITIN_HUMAN|ENSG00000155657|MXE2|1096|chr2|178682903|178683291|−2|r762|T1,sp|Q8WZ42|TITIN
RVEAEPAEEVTIMEEK q value: 2.9253e−05 Tr_novel:TRUE RefSeq_Novel:TRUE
Search result spec prec mz: 620.9767 Actual spec prec mz: 620.97662
Fragments matched per AA: 1.88 Proportion of top 20 peaks matched: 0.55
Intensity
6e+05
4e+05
2e+05
0e+00
250
500
750
1000
M/Z
b5
y6
b7
y4
b8
b9
y1
y2
y9
y5
y7
y8
b4
b6
y3
y10
b10
y11
B
Scatterplot of predicted elution time
Fitting R2: 0.87
Novel peptide residual Z score: −0.174
Number of peptides: 2407
Pred. hydrophobicity
50
25
0
0
25000
50000
75000
Experimental scan number
C
Distributions of residuals from best−fit line
of predicted RT vs Expt. scan number
Line: Z score of novel peptide
Z: −0.174
count
600
400
200
0
−10
−5
0
5
10
Z scores of residuals from best linear fit
